# Identification of Multigene Biomarker for Shrimp White Feces Syndrome by Full-Length Transcriptome Sequencing

**DOI:** 10.3389/fgene.2020.00071

**Published:** 2020-02-18

**Authors:** Shenzheng Zeng, Renjun Zhou, Shicheng Bao, Xuanting Li, Zhixuan Deng, Dongwei Hou, Shaoping Weng, Jianguo He, Zhijian Huang

**Affiliations:** ^1^State Key Laboratory of Biocontrol, Southern Marine Sciences and Engineering Guangdong Laboratory (Zhuhai), School of Marine Sciences, Sun Yat-sen University, Guangzhou, China; ^2^South China Sea Resource Exploitation and Protection Collaborative Innovation Center, Sun Yat-sen University, Guangzhou, China

**Keywords:** pacific white shrimp, white feces syndrome, multi-gene biomarker, full-length transcriptome sequencing, metagenomic sequencing, qPCR

## Abstract

The pacific white shrimp, *Litopenaeus vannamei*, with the largest shrimp industry production in the world, is currently threatened by a severe disease, white feces syndrome (WFS), which cause devastating losses globally, while its causal agents remain largely unknown. Herein, compared to the Control shrimp by metagenomic analysis, we firstly investigated that the altered functions of intestinal microbial community in WFS shrimp were the enrichment of bacterial chemotaxis and flagellar assembly pathways, hinting at a potential role of pathogenic bacteria for growth and development, which might be related to WFS occurrence. Single-molecule real-time (SMRT) sequencing was to further identify the gene structure and gene regulation for more clues in WFS aetiology. Totally 50,049 high quality transcripts were obtained, capturing 39,995 previously mapped and 10,054 newly detected transcripts, which were annotated to 30,554 genes. A total of 158 differentially expressed genes (DEGs) were characterized in WFS shrimp. These DEGs were strongly associated with various immune related genes that regulated the expression of multiple antimicrobial peptides (e.g., antilipopolysaccharide factors, penaeidins, and crustin), which were further experimentally validated using quantitative PCR on transcript level. Collectively, multigene biomarkers were identified to be closely associated with WFS, especially those functional alterations in microbial community and the upregulated immune related gene with antibacterial activities. Our finding not only inspired our cogitation on WFS aetiology from both microbial and host immune response perspectives with combined metagenomic and full-length transcriptome sequencing, but also provided valuable information for enhancing shrimp aquaculture.

## Introduction

Aquaculture remains an important source of food and nutrition for millions of people worldwide, which plays an essential role in meeting the urgent global food demand (Low et al., 2017). The pacific white shrimp, *Litopenaeus vannamei*, with the largest production by tonnage in the global shrimp industry (>4.1 million tons per year) (FAO, 2019), is currently facing challenges due to several diseases. White feces syndrome (WFS) is one of the most severe disease, which has caused destructive losses in cultured shrimp farming (Tangprasittipap et al., 2013; Hou et al., 2018; Xiong, 2018). Some progresses has been made to toward understanding the occurrence of WFS: there is a close relationship between dysbiosis of the intestinal microbiota (IM) dysbiosis and WFS (Xiong et al., 2017; Hou et al., 2018). It has been reported that the intestinal bacterial communities of shrimp with WFS exhibit less diversity but are more heterogeneous than those of healthy shrimp, and the intestinal bacterial communities of WFS shrimp and healthy shrimp differ significantly (Hou et al., 2018). Similar results were observed in another study that several distinguished taxa were responsible for WFS, and growth of candidate pathogens contributed to the altered intestinal microbial interaction (Xiong et al., 2018). These studies focused on the interplay between shrimp health and IM dysbiosis. The puzzle about how the shrimp responds to IM alteration remains largely unknown. This insufficiency can be partially attributed to the lack of a sequenced shrimp host genome and genetic regulation. Given that the aetiology of WFS is uncertain, the discovery of shrimp multigene biomarker for identification of WFS should be a major goal of research efforts.

Identification of biomarkers through transcriptome analysis, which covers both coding and noncoding RNA and quantifies gene expression, exhibits the full information about all RNA molecules transcribed by the genome under a certain set of physiological or pathological conditions, especially disease conditions (Mutz et al., 2013). Also, it provides the first steps toward functional characterization and annotation of genes, and it produces molecular fingerprints of disease processes (Morozova et al., 2009). For example, a succession of studies using the second-generation RNA-Seq to evaluate the potential mechanism and probable markers in aquatic animal diseases has been performed in recent years (Morozova et al., 2009; Stockhammer et al., 2010; Ordas et al., 2011; Zhang et al., 2011; Ribas et al., 2019). The role of innate immune response to bacterial evasion in zebrafish embryos was determined by transcriptome analysis, which also indicated a series of differentially expressed genes (DEGs) linked to the immune response activities (Stockhammer et al., 2010). In addition, another investigation into infection of catfish with the pathogen *Edwardsiella ictaluri* identified several biomarkers that played important roles in catfish mucosal immune response (Li et al., 2012). Biomarkers contributing to the genetic resistance of the yellow catfish to bacterial infections were also established using transcriptome analysis (Zhu et al., 2017). Similarly, prior studied used RNA-seq to uncover the transcriptome changes of *Penaeus monodon* that suffered from white spot syndrome virus and acute hepatopancreatic necrosis disease (Mohd Ghani and Bhassu, 2019; Soo et al., 2019). However, the research data cited above were generated by the second-generation sequencing technology, which meant that short-read sequence prevented the assembly of long transcripts (Xu et al., 2015). To overcome this deficiency, single-molecule real-time (SMRT) sequencing carried out in a PacBio system provides a third-generation sequencing platform that is widely applied due to its long reads (Chaisson et al., 2015). In comparison with paired-end sequencing technology, the methodological advantages of SMRT sequencing mainly include better completeness for long-sequence molecules and higher accuracy to identify alternative isoforms (Chen et al., 2017). The use of SMRT sequencing offers access to more complete full-length transcriptome data, as has been recently reported (Sharon et al., 2013; Dotterweich et al., 2016). For instance, a study about the full-length transcriptome of diabetic pig models showed multiple genetic alterations that could contribute to diabetic diseases (Zhang et al., 2019). However, the method of PacBio long-read sequencing could not be directly used to quantify gene expression, which still need RNA-seq for quantitative analysis of transcripts (Chen et al., 2017; Jia et al., 2018). Therefore, with the aim of exhibiting the transcriptome-wide landscape and identifying multigene markers for WFS, both SMRT and RNA-seq were employed to compare the healthy and WFS shrimp.

In the present study, for better identifying the metagenomic features of WFS, IM alterations associated with WFS were evaluated using metagenome analysis. Full-length transcriptome sequencing was then conducted to identify multigene biomarkers for WFS, and these biomarkers were confirmed to be involved in shrimp antimicrobial immune activity. Collectively, these valuable findings improve our understanding of the microbe-host interaction in shrimp, and provide clues regarding the aetiology of WFS, which could facilitate the prevention and treatment of WFS in shrimp aquaculture.

## Results

### Alterations of IM Diversity and Metagenome in WFS

In total, there were 68.9 Gb paired-end reads generated from seven Control and six WFS shrimps, with an average of 5.3 ± 0.83 million reads per sample. For each sample, high-quality sequencing reads (84.95%–85.80%) were *de novo* assembled into long scaffolds, which were used for taxonomic classification and functional annotation.

**Figure 1:**
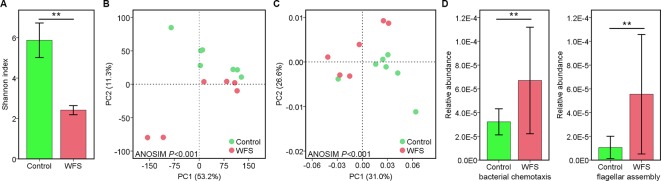
Identification of the microbial alterations in white feces syndrome (WFS). **(A)** The *α*-diversity comparison between Control (n = 7) and WFS (n = 6) groups. Shannon index, *P* = 0.003 (Student's *t*-test); **(B)** Samples were clustered into two group by PCoA using Bray-Curtis distance. The microbial composition differed significantly between Control and WFS groups. **(C)** Comparative analysis of microbial gene functions. Principal coordinate analysis (PCoA) based on the relative abundance of all Kyoto Encyclopedia of Genes and Genomes (KEGG) orthology groups. **(D)** Comparison of the relative abundance of bacterial chemotaxis and flagellar assembly pathways between Control and WFS groups. Each bar represents the mean ± SD of the samples. Significant differences are indicated by asterisks (**, *P* < 0.01).

A total of 1,258 taxa were classified across the enrolled seven Control (no clinical signs) and six WFS samples. The *α*-diversity in WFS was significantly lower than that in Control, as supported by the Shannon index (*P* < 0.001) (**Figure 1A**). To explore the difference between the microbial communities of the Control and WFS groups, a principal coordinate analysis (PCoA) as performed using Bray-Curtis distance. It revealed that the bacterial communities in WFS were notably different from those in the Control group (**Figure 1B**), which was confirmed by analysis of similarities (ANOSIM, *P* < 0.001).

Using the Kyoto Encyclopedia of Genes and Genomes (KEGG) database, the IM functions were evaluated across groups. The PCA based on genes demonstrated marked differences in IM functional structures between two groups (ANOSIM, *P* < 0.001) (**Figure 1C**). Fourteen KEGG pathways, including bacterial chemotaxis, caprolactam degradation, lysosome and flagellar assembly increased significantly in WFS (*P* < 0.05) compared with Control (**Figure 1D**). Additionally, the correlation between compositional and functional structures was evaluated using Mantel test, which revealed that these two structures were significantly correlated (*P* < 0.001, **Table 1**). These results demonstrated the close correlation between IM alterations and WFS occurrence.

**Table 1:** Mantel test the correlation between compositional and functional structures.

	permutations	r value	*P* value
Functional vs. Compositional structures	999	0.418	0.001

### Assembly of the Full-Length Transcriptome of Shrimp Intestine

**Figure 2:**
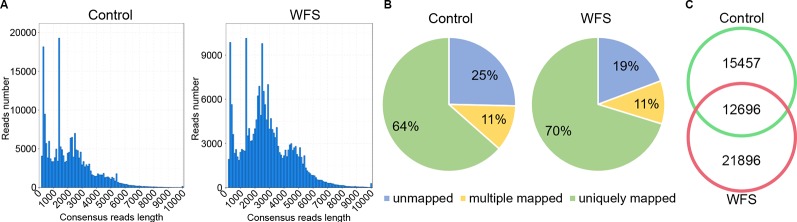
Sequencing information of full-length transcriptome. **(A)** The length distribution of consensus reads in Control and white feces syndrome (WFS). **(B)** The consensus reads were aligned to the shrimp reference genome. More than 75% reads have been mapped to the shrimp genome. **(C)** Veen diagram showed the overlap of detected transcripts between Control and WFS.

For better annotation of the shrimp transcriptome, two cDNA libraries covering molecules of different lengths (A: 1–4 kb; B: > 4 kb) were generated and sequenced on the PacBio platform, using mixed RNA samples derived from shrimps in the Control and WFS groups. A total of 899,413 and 921,477 polymerase reads were obtained from the Control and WFS groups (**Table 2**), which were then clustered into 768,024 and 719,214 circular consensus sequence (CCS) (**Table 2**). The CCS reads were classified into 414,157 and 478,319 full length nonchimeric reads (FLNS) in Control and WFS, respectively. These FLNS reads were subjected to redundancy removal, producing 215,082 and 212,308 high-quality consensuses for each group (**Table 2**), and the length distribution was shown in **Figure 2A**. After further correction using Illumina RNA-seq reads (**Table 2**), they were mapped to the shrimp genome (**Figure 2B**), 28,153 and 34,592 mapped transcripts in the genome were obtained in the Control and WFS, respectively. There were 12,696 transcripts common to both the Control and WFS groups (**Figure 2C**), while the unique transcripts were 15,457 and 21,896 in the Control and WFS groups, respectively.

**Table 2:** Sequencing information of full-length transcriptome.

Sample	Polymerase reads	CSS number	FLNC number	Consensus number	N50 before correction	N50 after correction
Control	899,413	768,024	414,157	215,082	2,976	2,976
WFS	921,477	719,214	478,319	213,308	3,552	3,552

### Transcriptome Difference Between Control and WFS

**Figure 3:**
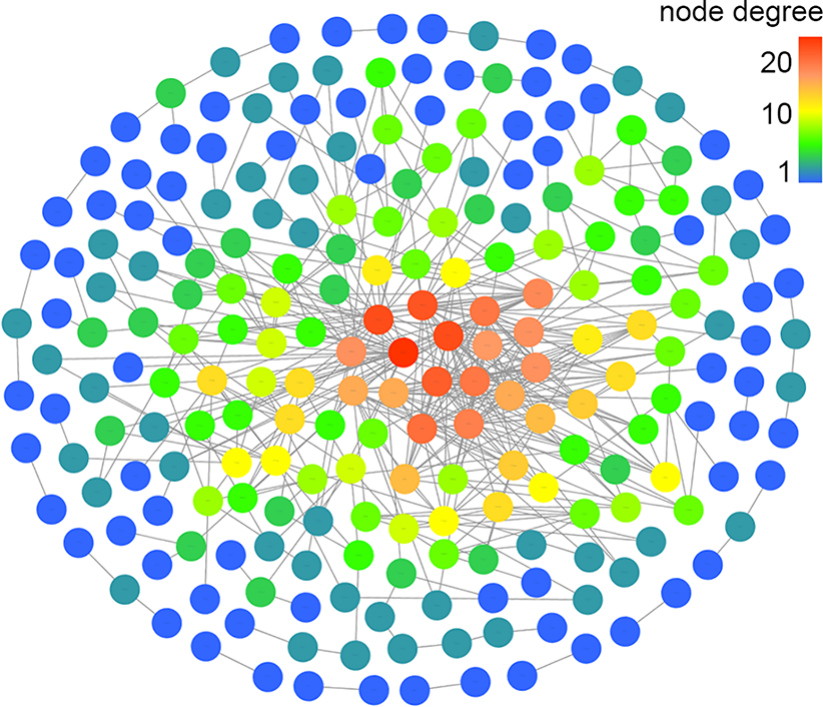
Correlation network of the shrimp transcriptome. The relationship among all genes was estimated by Spearman's correlation analysis. And those with low correlated (|r| < 0.7) are not shown.

**Figure 4:**
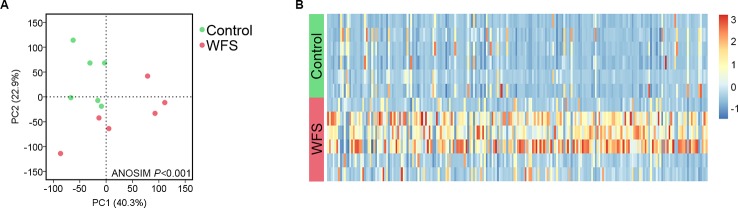
Comparative analysis of transcript profiles between Control (n = 6) and white feces syndrome (WFS) (n = 6). **(A)** The principal coordinate analysis (PCoA) based on the expression level of all transcripts. The WFS samples were distinct from the Control samples. **(B)** A total of 206 transcripts were identified to be differed significantly between Control and WFS. The heatmap was conducted based on the FPKM value of each transcripts.

Based on the mapped transcripts, network analysis was conducted to understand the complex gene regulatory network of shrimp intestine (**Figure 3**). The nodes with the greatest number of neighbors were identified (**Table 3**), which could be considered significant hubs of the network. Some important keystone genes, such as ALF-D, *Crustin*A and *Lv*serpin (Nigou et al., 2013; Liu et al., 2014; Barreto et al., 2018), have been reported to play central roles in antimicrobial activities. PCoA was performed to determine the transcript profiles across groups. Results showed that samples from different groups were separate (ANOSIM, *P* < 0.001) (**Figure 4A**). DEGs between the Control and WFS groups were identified from combined data of PacBio transcriptome and Illumina transcriptome. The alterations involved a total of 206 transcripts that differed significantly between the Control and WFS groups (**Figure 4B**), including 33 transcripts that were upregulated in Control shrimp and 173 transcripts that were downregulated in WFS shrimp. These 206 transcripts were also compared with known shrimp genes using BLASTN, which showed that 206 transcripts were identified involving 157 genes. Their functional profiles are shown in **Supplementary Table 1**.

**Table 3:** The transcript hubs (nodes with the most degrees) information of the correlation network.

Transcript ID	Node degree	Description
Novelgene1822_novel03	26	*Litopenaeus vannamei* single VWC domain protein 4
XM_027372426.1	23	*Litopenaeus vannamei* peroxinectin
LOC113828743_novel01	23	*Litopenaeus vannamei* prophenoloxidase-1
Novelgene4975_novel01	22	*Litopenaeus vannamei* serine proteinase inhibitor (SERPIN)
XM_027357402.1	21	*Litopenaeus vannamei* serine proteinase inhibitor (SERPIN)
XP_027235787.1	19	*Litopenaeus vannamei* prophenoloxidase-1
XM_027351129.1	18	*Litopenaeus vannamei* anti-lipopolysaccharide factor AV-K isoform
XM_027382194.1	18	*Litopenaeus vannamei* dorsal
XP_027206930.1	17	*Litopenaeus vannamei* anti-lipopolysaccharide factor AV-K isoform
LOC113800785_novel03	16	*Litopenaeus vannamei* dicer 2
LOC113815940_novel02	15	*Litopenaeus vannamei* fatty acid synthase (FAS)
Novelgene0238_novel02	13	*Litopenaeus vannamei* penaeidin 2b
XM_027373623.1	13	*Litopenaeus vannamei* crustin A
XM_027357401.1	12	*Litopenaeus vannamei* serine proteinase inhibitor (SERPIN)
LOC113830391_novel01	11	*Litopenaeus vannamei* hypoxia inducible factor 1 beta
Novelgene1703_novel02	10	*Litopenaeus vannamei* caspase 3

**Figure 5:**
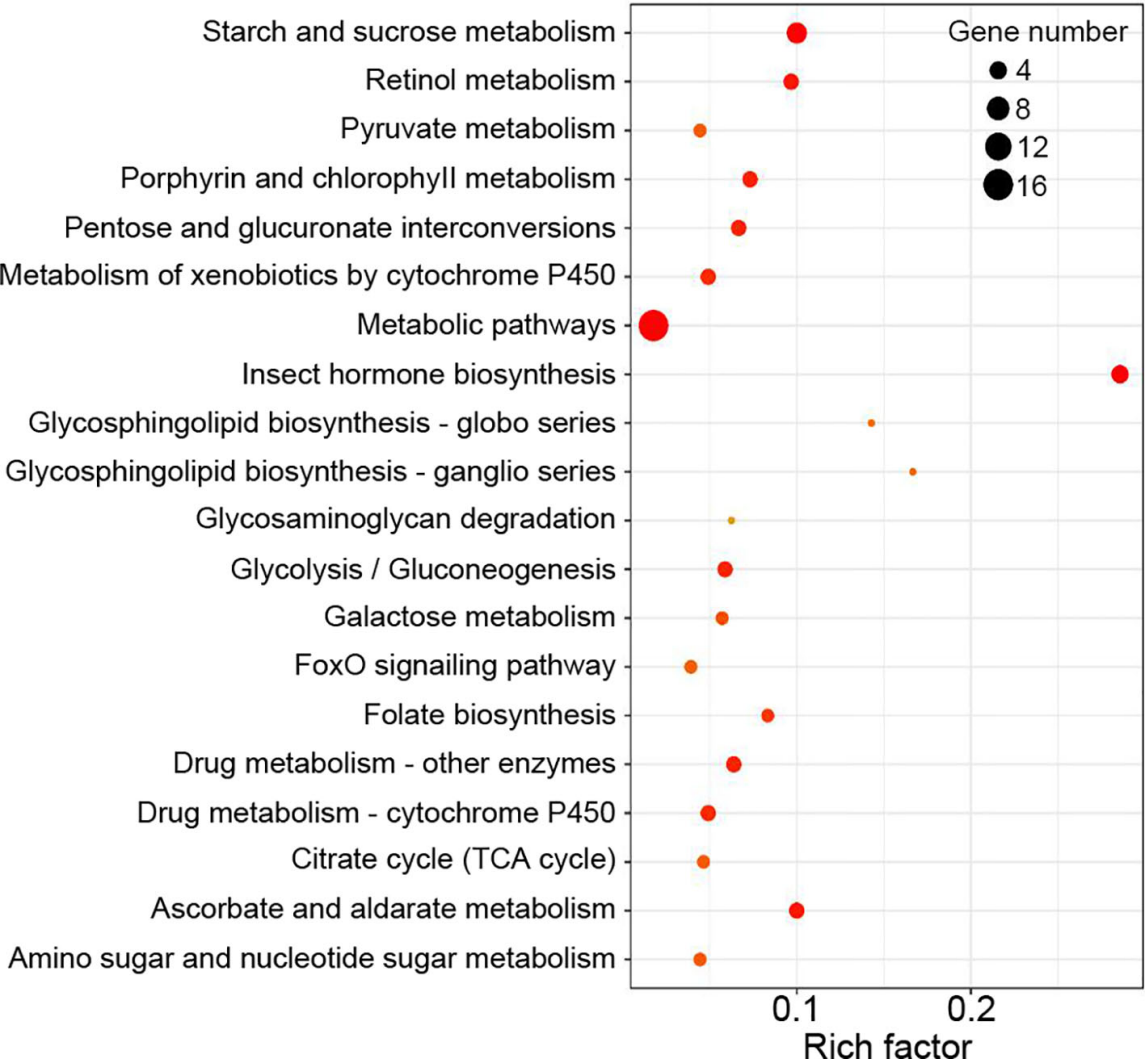
Kyoto Encyclopedia of Genes and Genomes (KEGG) enrichment analysis. The KEGG pathways were considered significantly enriched by the differentially expressed genes (DEGs) expressions in different groups.

Functional enrichment analysis showed that DEGs between the Control and WFS groups were associated with 34 KEGG pathways, including starch and sucrose metabolism, metabolic pathways, retinol metabolism, glycolysis-gluconeogenesis and lysosome (**Figure 5**), which were reported to be linked to pathogen interaction (Wu et al., 2019).

### Identification of Genes-Based Markers of WFS

**Figure 6:**
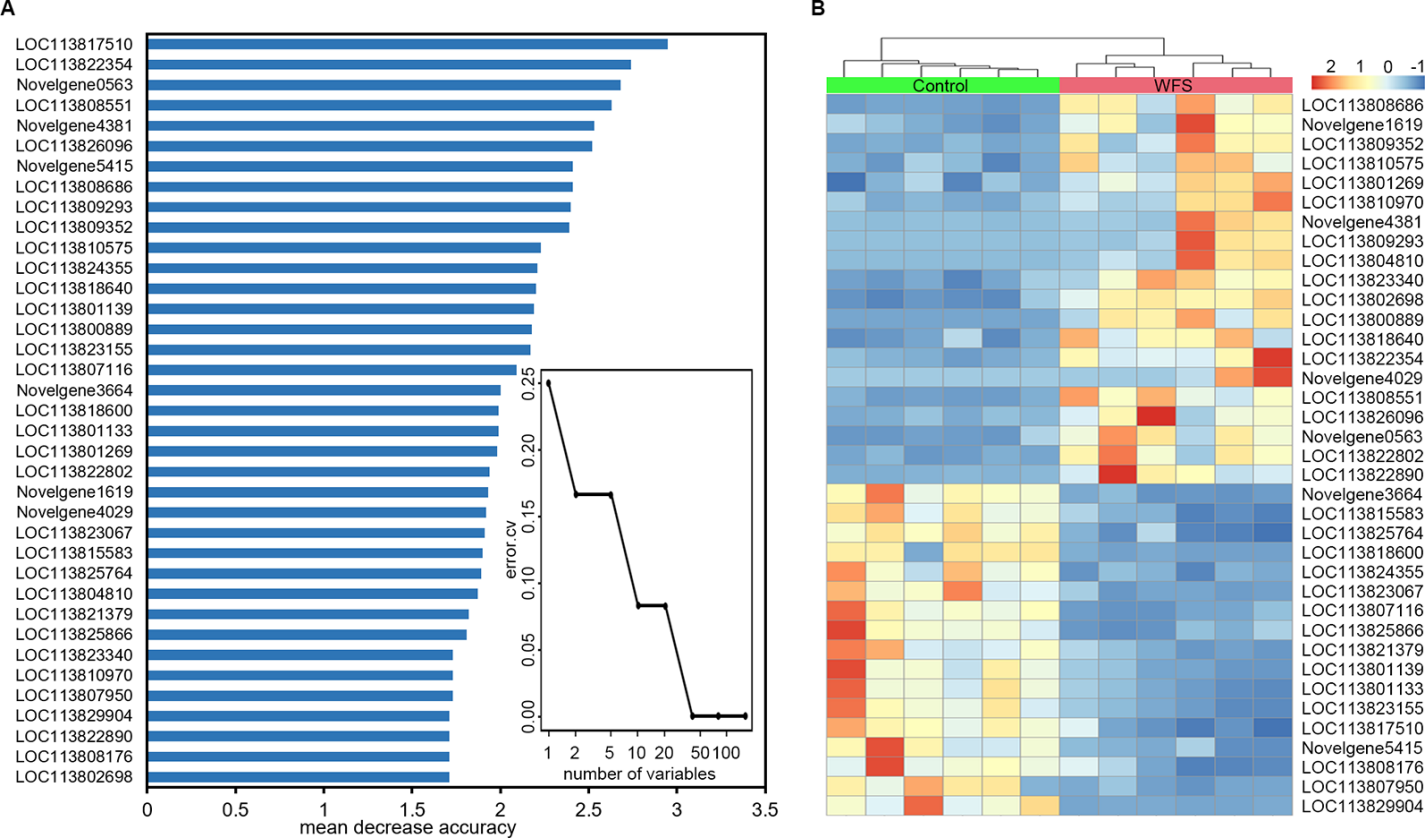
Identification of predictive biomarkers for white feces syndrome (WFS) by random forests model. **(A)** The 10-fold cross-validation on a random forests model suggested a total of 37 genes were selected as the optimal markers. The 37-top ranked biomarkers were shown according to the mean decrease accuracy. **(B)** Heatmap based on the 37 markers revealed that the WFS group was clearly distinct to the Control group.

To identify a minimum predictive gene biomarker that could be used to distinguish WFS from Control, predictive performance was evaluated using 10-fold cross-validation with a random forest model. The features were selected according to the 157 significantly altered genes on the training samples to reduce bias through overfitting and to assure independence in the test data. Results suggested 37 genes as the optimal gene-marker set for WFS (**Figure 6A**). The 37-top ranked WFS-discriminatory genes by variable importance measures are presented in **Figure 6A**. Using the profiles of these gene-markers as independent variables, the simplified random forest model was used to predict health status across all samples, which successfully exhibited a high predictive performance (100% accuracy) (**Table 4**). Moreover, the express level of the 37 gene markers biomarkers were exhibited using a heatmap (**Figure 6B**), which revealed that the WFS group was clearly distinct to the Control group, suggesting that the identification with random forest model was efficient.

**Table 4:** The predictive accuracy based on the 37-top biomarkers for white feces syndrome (WFS) using the random forests model.

	Control	WFS	Class. error(%)
Predicted as Control	6	0	0
Predicted as WFS	0	6	0
Overall accuracy			100

**Figure 7:**
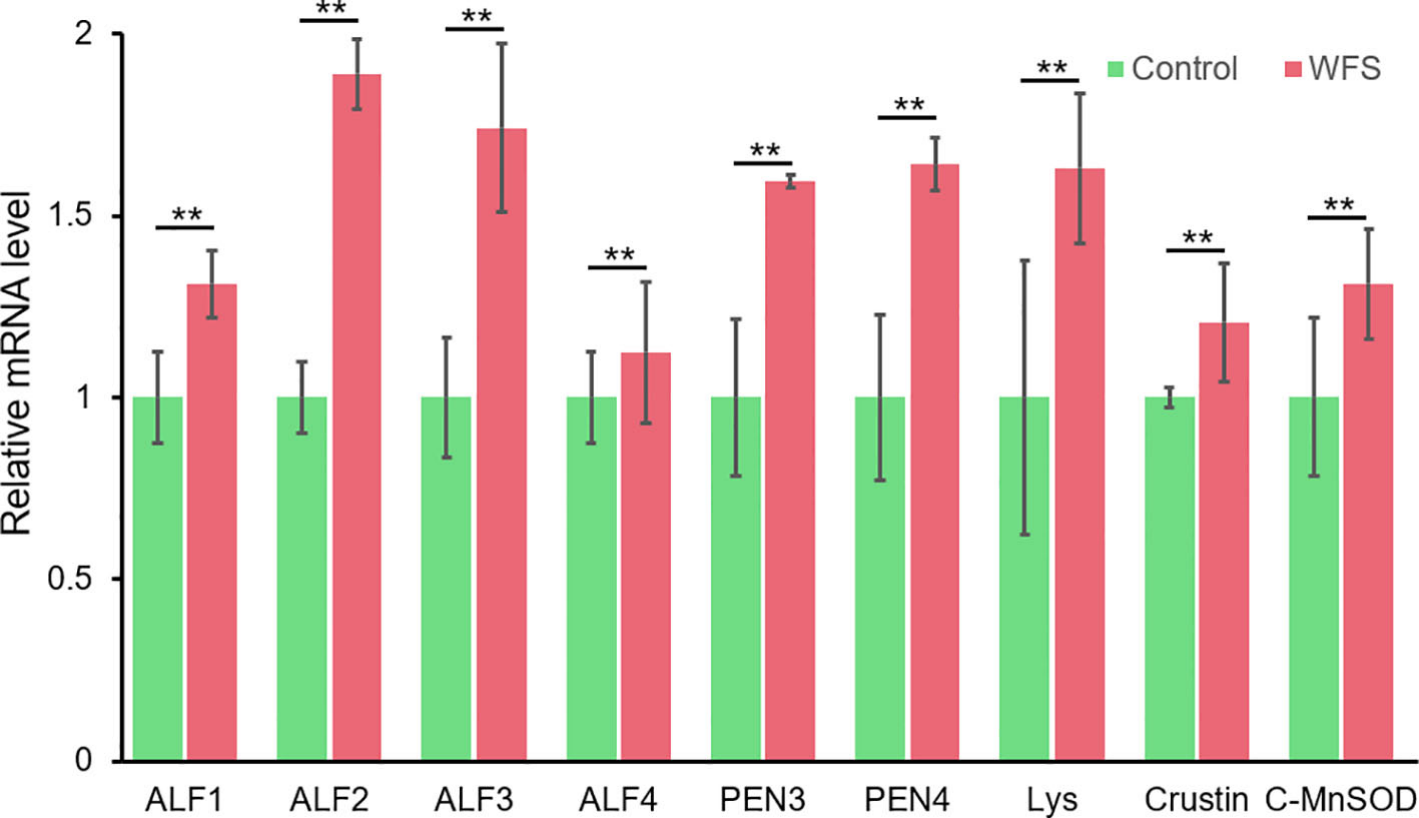
Comparison of the expression of various antimicrobial related genes between Control and white feces syndrome (WFS). Each bar represents the mean ± SD of the samples. Significant differences are indicated by asterisks (**, *P* < 0.01).

Notably, nine altered genes were responsible for antimicrobial activity in shrimp (**Table 5**). To primarily investigate the probability that these genes could be used as markers for WFS, the expression lever of these genes in intestine was detected using real-time PCR (**Figure 7**). The levels of expression of antimicrobial genes [antilipopolysaccharide factors (ALFs), penaeidins (PENs), lysozyme, crustin and cytosolic manganese superoxide dismutase (C-MnSOD)] were significantly upregulated in WFS relative to Control. The key gene biomarkers involved in shrimp antimicrobial immunity was upregulated in WFS, corroborating the potential of genes to serve as biomarkers for detecting WFS in shrimp.

**Table 5:** The altered genes that were responsible for antimicrobial activity in shrimp.

Gene name	Description
LvPxt	*Litopenaeus vannamei* peroxinectin
Lvserpin	*Litopenaeus vannamei* serine proteinase inhibitor (SERPIN)
LvproPO1	*Litopenaeus vannamei* prophenoloxidase-1
LitvanALF-B	*Litopenaeus vannamei* anti-lipopolysaccharide factor AV-K
LitvanALF-D	*Litopenaeus vannamei* anti-lipopolysaccharide factor AV-K
LvPen2	*Litopenaeus vannamei* penaeidin 2b
LvPPAE2	*Litopenaeus vannamei* prophenoloxidase activating enzyme 2
LitvanALF-C	*Litopenaeus vannamei* anti-lipopolysaccharide factor AV-K
LvSVC5	*Litopenaeus vannamei* single VWC domain protein 5

## Discussion

Studies on the shrimp IM-disease relationship have identified long lists of implicated microbes that may reflect biomarkers of disease (Xiong et al., 2017; Dai et al., 2018; Hou et al., 2018), without clearly elucidating either the altered microbial functions or the host response against pathogens (Xiong, 2018). In the present study, we identified predictive multigene biomarkers for WFS by combining metagenome and full-length transcriptome data. This work partially meets the urgent need for diagnostic biomarkers for the detection of diseases in shrimp, which also offers insights into the WFS aetiology from both microbial and host immune angles.

Microbial diversity, especially, IM diversity, may be considered a biomarker of host health (Ren et al., 2018). Similarly, our study showed that the α-diversity of IM was significantly less pronounced in WFS (**Figure 1A**), providing more clues to establish that the beta-diversity may be associated with disease. In our previous work (Hou et al., 2018) and other related studies (Xiong et al., 2017; Dai et al., 2018), a series of complicated taxa were found to be associated with WFS by comparing the microbial compositional differences between healthy and diseased shrimp. However, the functional differences between Control and WFS shrimp still remain unclear. To fill in the blank area, we observed an alteration in bacterial gene function structure, together with the divergence of functional pathways, such as bacterial chemotaxis and flagellar assembly (**Figure 1**). The bacterial chemotaxis and flagellar assembly modules, which were involved in guiding bacterial pathogens to find suitable colonization sites for growth and development, can be indicative of many types of bacterial infectious diseases (Stocker and Seymour, 2012). Thus, these findings suggest that alterations in these functional genes might have a strong link to WFS.

Transcriptome analysis, which can be used to improve understanding of the genes underlying host response to disease, can also facilitate the identification of biomarkers for specific diseases as reported in compelling studies (Stockhammer et al., 2010; Zhu et al., 2017; Klett et al., 2018). A prior study identified three genes as new potential candidates for white spot syndrome virus resistance markers in giant tiger shrimp using transcriptome analysis (Mohd Ghani and Bhassu, 2019). One previous study identified a total of 24 key genes involved in multiple KEGG signaling pathways in Japanese flounder infected with *Edwardsiella* by sequencing the gill transcriptome and analyzing the gene interaction network (Li et al., 2018). Another study utilized RNA-seq to profile the 1,475 DEGs in Nile tilapia suffering from *Streptococcus agalactiae* infection, which suggested immune activation and inflammation in the host response (Wang et al., 2016). In the present study, we first established that the transcript-profile in WFS differed significantly from that of the Control, and then identified 157 DEGs by comparing Control and WFS samples (**Figure 4**). The optimal 38 biomarkers indicated by the 157 DEGs were identified by random 10-fold cross-validation, and the predictive random forest model successfully achieved an accurate classification potential for distinguishing WFS samples from Control samples (**Figure 6**). This work was the first attempt to distinguish the alterations of WFS at the transcript level. Our findings also suggested that transcriptome-targeted biomarkers might be suitable for identification of WFS.

Furthermore, we found that multigene biomarkers were involved in antimicrobial activities. Genes related to three kinds of antimicrobial peptides (ALFs, PENs, and crustin) were upregulated in WFS, and this was further validated by qPCR (**Figure 7**). The ALFs, which could recognize the invasions of Gram-negative bacteria, are here described as highly cationic polypeptides with a broad spectrum of potent antimicrobial activities in marine crustaceans (Nigou et al., 2013). In pacific white shrimp, the ALFs form a diverse family that have been proved to be essential in the protection of shrimp against different microbial infections (such as *Vibrio* and *Aeromonas*) (de Lorgeril et al., 2008). The penaeidins are a family of antimicrobial peptides that participate in host defense reactions against invading microbes (Destoumieux et al., 1999). Unlike ALFs, the antibacterial activities of PENs are essentially directed against Gram-positive bacteria (Destoumieux et al., 1999). Crustins form a large family of antimicrobial peptides, exhibiting protease inhibitory properties and regulatory functions in bacterial growth to all microbes (Barreto et al., 2018). The C-MnSOD and lysozymes are immune effector proteins that have antibacterial activities (Niu et al., 2018). Generally, the upregulation of antimicrobial peptides and immune effector genes in WFS has provided clues to understand the close relationship between IM alterations and WFS.

Based on our results and current knowledge, we take the following three perspective into consideration for WFS: (I) the IM composition and diversity were found to differ significantly between Control and WFS shrimp, as shown in previous studies (Dai et al., 2018; Hou et al., 2018); (II) microbial functions also differed between Control and WFS, which indicated a potential role of pathogenic bacteria for growth and development (**Figure 1**); (III) the host upregulated genes were involved in multiple antimicrobial activities (**Figure 6**). From the microbial and host immune angles, all the findings suggested that the IM alterations were relevant to WFS, which might guide us into understanding the pathogenesis of this disease in shrimp, and focus on the infection processes through the full-length transcriptome data.

In summary, we have clearly described the altered profiles of microbial functions, exhibited the significant differences in host transcriptome, and finally identified multigene biomarkers for WFS. These findings may improve our understanding of the development of WFS with multiomics data and provide valuable biomarkers with strong power for WFS prediction.

## Materials and Methods

### Sample Collection

Shrimp with an average length of 11 cm were collected in Zhuhai, Guangdong, China (22.38°N, 113.23°E), in 2018. A total of six healthy shrimp (Control group) and six shrimp exhibiting WFS (WFS group) were included in this study to compare the IM metagenome and host transcriptome between the two statuses. The surfaces of the shrimp were sterilized with 70% ethanol, and their intestines were aseptically dissected on ice. All intestine samples were stored at −80°C before nucleotide extraction.

### Metagenomic Sequencing and Gene Catalog Construction

The genomic DNA was extracted by the QIAamp PowerFecal DNA Kit (Qiagen, Germany). Sequencing libraries were generated using the NEBNext Ultra DNA Library Prep Kit for Illumina (New England Biolabs, UK), and index codes were added to attribute sequences to each sample. The libraries were sequenced by Illumina Hiseq2500 platform in MAGIGENE Co., Ltd. (Shenzhen, China).

Quality control was conducted by Trimmomatic software (Version 0.38) (Bolger et al., 2014). The reads aligned to the NCBI nonredundant (NR) database were removed with MEGAHIT software (https://github.com/voutcn/megahit, Version 1.05). The remaining high-quality reads were used for further analysis. The assembly of reads was executed using MEGAHIT *de novo*. For each sample, a series of k-mer values (49 to 87) were used, and the optimal series with the longest N50 value were chosen for the remaining scaffolds. The clean data were mapped against scaffolds using MEGAHIT. Genes were predicted on scaftigs longer than 500 bp using Prodigal software (Version 2.6.3) (Hyatt et al., 2010). Then, a nonredundant gene catalog was constructed with Linclust software (Version 2.0) (Steinegger and Soding, 2018), using a sequence identity cut-off of 0.9. To determine the abundance of genes, reads were realigned to the gene catalog with BBMap software (https://sourceforge.net/projects/bbmap/, Version 37.68). The abundance of genes was calculated by counting the number of reads and normalizing by gene length.

### Transcriptome Sequencing and Data Processing

The shrimp intestine RNA was extracted using the RNeasy Mini Kit (Qiagen). To prepare the library for full-length transcriptome sequencing, RNA samples of the same group were pooled at equimolar concentrations. The library was conducted using the Clontech SMARTer PCR cDNA Synthesis Kit and the BluePippin Size Selection System (Pacific Biosciences, US) and was sent for sequencing by the SequelII platform (Pacific Biosciences). The library for Illumina sequencing was generated using Collibri Stranded RNA Library Prep Kits for Illumina Systems (ThermoFisher) and was sequenced by the HiSeq2500 platform (Illumina, US), which was conducted by Novogene Bioinformatics Technology Co., Ltd. (Beijing, China).

Sequence data were processed using the SMRTlink 5.0 software (https://www.pacb.com/support/software-downloads/, Version 6.0). Circular consensus sequence (CCS) was generated from subread BAM files, which were then classified into full length and nonfull length reads. Full length and nonfull length fasta files were fed into the cluster step, which does isoform-level clustering, followed by final polishing. Additional nucleotide errors in consensus reads were corrected using the Illumina RNA-seq data with the LoRDEC software (Version 0.7) (Salmela and Rivals, 2014). To map the reads to the pacific white shrimp reference genome (NCBI Genome database ID: 10710), GMAP software (Version 2017-06-20) was used to align consensus reads (Wu and Watanabe, 2005). For quantification of gene expression level, HTSeq software (Version 0.6.1) was used to count the reads mapped to each gene (Anders et al., 2015). Then the expected number of fragments per kilobase of transcript sequence per Millions base pairs sequenced (FPKM) of each gene was calculated based on the length of the gene and number of reads mapped to this gene.

### Real-Time PCR

Total RNA was reverse transcribed into cDNA using a PrimeScript RT Reagent Kit (Takara, Japan). Real-time PCR was performed with SYBR Premix Ex Taq™ II (Takara, Japan) and 500 nM of each primer on a LightCycle 480 System (Roche, Germany). The levels of expression of genes were determined using the 2^−ΔΔCt^ method after normalization to the internal control gene elongation factor 1 alpha (EF1-α). Sequences of primers used in this study are listed in **Supplementary Table 2**.

### Statistical Analysis

To evaluate the differences between groups, statistical analysis was conducted using SPSS (Version 21.0). If data were normally distributed, a two-tailed Student's *t* test was used to determine significance in experiments with two groups. In cases where data were not normally distributed, Welch's test was performed. Beta-diversity comparison based on Bray-Curtis distance was accomplished using vegan package in R software (https://www.r-project.org/, Version 3.6.0). Random forests regression was used to regress relative abundances of taxa in the temporal profiles of Control and WFS groups, using default parameters in R (randomForest package, ntree = 5000). Mantel Test and ANOSIM was calculated by Vegan package in R. Spearman's rank correlation was conducted to assess the correlation of pairs of variables in SPSS.

## Data Availability Statement

The sequencing data used in this study are available in the NCBI Short Read Archive (https://www.ncbi.nlm.nih.gov/sra) under SAMN13354306.

## Author Contributions

SZ, JH, and ZH conceived this study. SZ and ZH developed the experimental strategies and sampling design. SZ, RZ, ZD, XL, SB, DH and SW performed the sample collections, nucleotide extraction, sequencing data analysis and qPCR experiments. SZ, JH, and ZH wrote the manuscript. All authors read and approved the final manuscript.

## Funding

This work was financially supported by the China Agriculture Research System (CARS-48); China-ASEAN Maritime Cooperation Fund, China-ASEAN Center for Joint Research and Promotion of Marine Aquaculture Technology; Guangdong MEPP Fund (NO.GDOE (2019) A21); the Guangzhou Science Technology and Innovation Commission Project (201510010071); the Guangdong Ocean and Fishery Bureau Project (20164200042090023).

## Conflict of Interest

The authors declare that the research was conducted in the absence of any commercial or financial relationships that could be construed as a potential conflict of interest.
