# Supplementary material for: Identification of Multigene Biomarker for Shrimp White Feces Syndrome by Full-Length Transcriptome Sequencing
Source: Front Genet. 2020 Feb 18;11:71. doi: 10.3389/fgene.2020.00071 (PMC7040362; doi:10.3389/fgene.2020.00071)
Supplement: Supplementary file 2 [file Table_2.docx]

**Supplementary Table 2** Summary of primers used in this study.

| Primers | Sequences (5’ to 3’) | Reference |
| --- | --- | --- |
| *Lv*EF-1α-F | CCTATGTGCGTGGAGACCTTC | (Niu et al., 2018) |
| *Lv*EF-1α-R | GCCAGATTGATCCTTCTTGTTGAC |  |
| *Lv*ALF1-F | GGATGTGGTGTCCTGGATGG |  |
| *Lv*ALF1-F | GCGTCGTCCTCCGTGATG |  |
| *Lv*ALF2-F | GCGAACAAACTCACTGGACTG |  |
| *Lv*ALF2-F | ACATGCGACCCTGGAATACAG |  |
| *Lv*ALF3-F | GACCTGTCCAACCCTGAGC |  |
| *Lv*ALF3-F | TCGCCTCCTCCTCCGTTATC |  |
| *Lv*ALF4-F | CCTGGTGGCACTCTTCGC |  |
| *Lv*ALF4-F | ACGGTGAAGCGGCACTTATG |  |
| *Lv*PEN2-F | CCAAGGCGAAGCGTACAG |  |
| *Lv*PEN2-R | CAATTGCGAGCATCTGAGAC |  |
| *Lv*PEN3-F | CTCCTGCGTCCGCCATG |  |
| *Lv*PEN3-R | GTGTAACCGCCCTTGTACAC |  |
| *Lv*PEN4-F | GCCCGTTACCCAAACCATC |  |
| *Lv*PEN4-R | AACAATCCCCGTATCTGAAGC |  |
| *Lv*Crustin-F | CACAACCTGTTCCAACGACTAC |  |
| *Lv*Crustin-R | ACCTGCGATCCGAAGAATGAG |  |
| CMnSOD-F | TTGCCGCTACGAAGAAGTTG |  |
| CMnSOD-R | AGAAGATGGTGTGGTTCAAGTG |  |
| *Lv*Lys-F | CGGACTACGGCATCTTCCAG |  |
| *Lv*Lys-R | TCATCGGACATCAGATCGGAAC |  |

**Reference**

Niu, S., Yang, L., Zuo, H., Zheng, J., Weng, S., He, J., et al. (2018). A chitinase from pacific white shrimp *Litopenaeus vannamei* involved in immune regulation. *Dev*. *Comp*. *Immunol*. 85, 161-169. doi: 10.1016/j.dci.2018.04.013.
